# Supplementary material for: A copula based topology preserving graph convolution network for clustering of single-cell RNA-seq data
Source: PLoS Comput Biol. 2022 Mar 10;18(3):e1009600. doi: 10.1371/journal.pcbi.1009600 (PMC8979455; doi:10.1371/journal.pcbi.1009600)
Supplement: S1 File — S1 Text: Overview of the datasets. S2 Text: Marker analysis with sc-CGconv. Table A: Marker genes identified from the clustering results with sc-CGconv. Fig A: Performance of GCN on networks created from four datasets: receiver operating characteristic (ROC) curve for the validation is given for four datasets (see Table 1 of the main text for ROC score). Fig B: Marker analysis using sc-CGconv. After clustering DE genes are identified using clustering of PBMC data with sc-CGconv and results of marker genes on ultra large PBMC datasets. Panel-A. 2D UMAP visualization of PBMC dataset with original cell annotations. Panel-B. 2D UMAP visualization of clustering results with sc-CGconv. Panel-C. visualization of 12 markers which are overlayed based on their expression –low (blue) to high (yellow)- on the reference PBMC UMAP plot. Fig C: Figure depicts the results of marker gene analysis on melanoma datasets. Panel-A. 2D UMAP visualization of melanoma data with original annotation. Panel B. 2D UMAP visualization of clustering results with sc-CGconv. Panel-C. visualization of 9 markers which are overlayed based on their expression –low (blue) to high (yellow). (PDF) [file pcbi.1009600.s001.pdf]

A topology preserving graph convolution network for clustering of single-cell RNA seq data

S1 Text: Overview of datasets

- Baron [1]: The dataset is invoked with inDrop, a droplet-based single-cell RNA-Seq method, to determine the transcriptomes of over 12,000 individual pancreatic cells from four human donors and two strains of mice. Cells could be divided into 15 clusters that matched previously characterized cell types: all endocrine cell types, including rare ghrelin-expressing epsilon-cells, exocrine cell types, vascular cells, Schwann cells, quiescent and activated pancreatic stellate cells, and four types of immune cells. It contains 20125 number of genes and 8569 number of cells with 8 cell types.
- Klein [2]: This dataset was generated by the droplet barcoding method with an average total read count of 20,033.40 reads in the expression matrix. A total of eight single cell data sets are submitted: 3 for mouse embryonic stem (ES) cells (1 biological replicate, 2 technical replicates); 3 samples following LIF withdrawal (days 2,4, 7); one pure RNA data set (from human lymphoblast K562 cells); and one sample of single K562 cells. The dataset contains 24175 number of genes and 2717 number of cells with 4 cell types.
- Melanoma [3]: The dataset describes the diversity of expression states within melanoma tumors, it is obtained freshly resected samples, dissagregated the samples, sorted into single cells and profiled them by single-cell RNA-seq., The dataset contains 19783 genes and 68579 cells with 14 cell types.
- PBMC [4]: It is downloaded from `https://support.10xgenomics.com/single-cell-geneexpression/datasets`. The data is sequenced on Illumina NextSeq 500 high output with 20,000 reads per cell. It contains approx ∼68k number of cell with 11 cell types.

The dataset description is given in Main paper Table 1.

S2 Text: Marker analysis with sc-CGconv

Conventional procedure of Scanpy is followed identify markers (DE genes) from the clustering results with sc-CGconv. Scanpy utilized wincoxon rank sum test to find out the significant ($p < 0.05$) DE genes for each cluster which are treated as marker genes. We took top 50 marker genes with their p-value threshold 0.05 on all four datasets.

We found that 19 marker genes from melanoma dataset , 12 marker genes from PBMC dataset, 8 marker genes from Baron dataset, and 4 marker genes from Klein dataset are biologically significant according to Cell Marker database. The list of biologically significant marker genes are given in Table A.

Figs B and C depict the results of clustering and marker gene analysis for PBMC and Melanoma datasets respectively.

Table A: Marker genes identified from the clustering results with sc-CGconv.

Dataset	cell type	markers (pubmed id)
Melanoma [3]	CD8 T cell	TNFRSF9 (28622514),KLRC4 (28622514), CXCL13 (28622514)
	CD4 T cell	CTSW (28457750), CD69 (28566371), LTB (28263960), CD4 (12000723)
	Regulatory T cell	IL32 (30093597), LAG3 (28929191), FCRL3 (25762785), TNFRSF18 (23929911), LAT2 (28622514)
	Naive T cells	CD7 (7539656)
	T helper1 (Th1) cell	IFNG (20868565), STAT4
	CD4+ memory T cells	CCR7 (28929596)
	NK cell	BCL11B
	B cell	CD79B (29230012)
	Megakaryocyte	CTSW (30093597)
PBMC [5]	Regulatory T cell	IL32 (30093597)
	CD8 T cell	CCL5 (30093597)
	NK cells	NKG7 (8458737), GNLY (12884856)
	Effector CD8+ memory T cell	GZMH (28622514)
	Plasmacytoid dendritic cells	GZMB (19965634)
	CD4+ cytotoxic T cell	CST7 (28622514)
	B cell	CD79A (11396639), CD37 (24952935)
	Monocyte derived dendritic cells	CST3 (19956698)
	Megakaryocyte progenitors	PPBP (27084257), PF4 (30645026)
Baron [1]	Alpha cell	PAX6 (24309898), CD81 (27881302)
	Acinar cell	CD24 (24309898), SPINK1 (24309898)
	Duct Cell	ANXA2 (24309898), CTSD (24309898)
	Endothelial cell	TIMP1 (24309898)
	Beta cell	GAD2 (24309898)
Klein [2]	Stem Cell	DDX4 (21034600), SOX2, CD133 (24309898)
	Mesenchymal cell	S100A4 (24309898) ,GSN (24309898)
	Germ Cell	Sl39a10 (24309898)
	Endothelial cell	CD31 (24309898)

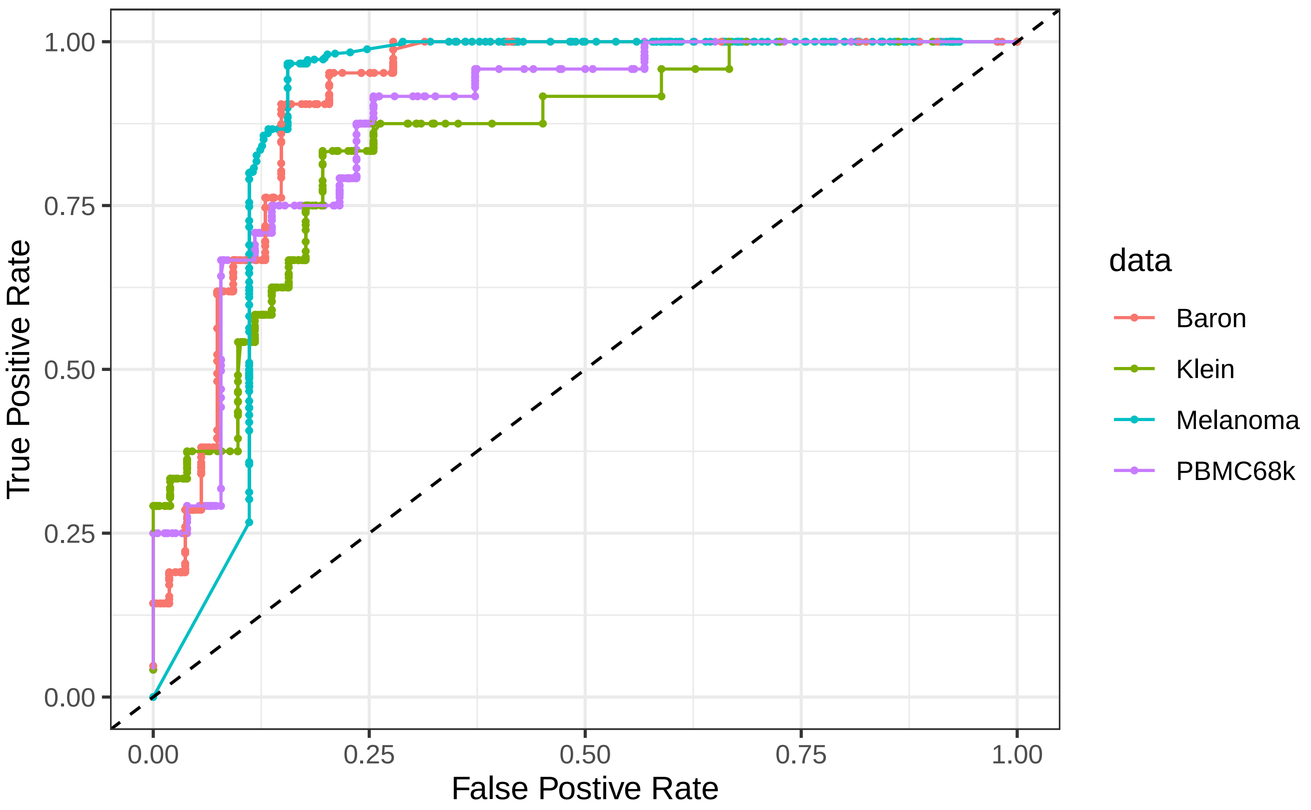

Fig A: Performance of GCN on networks created from four datasets: receiver operating characteristic (ROC) curve for the validation is given for four datasets (see table-1 of the main text for ROC score).

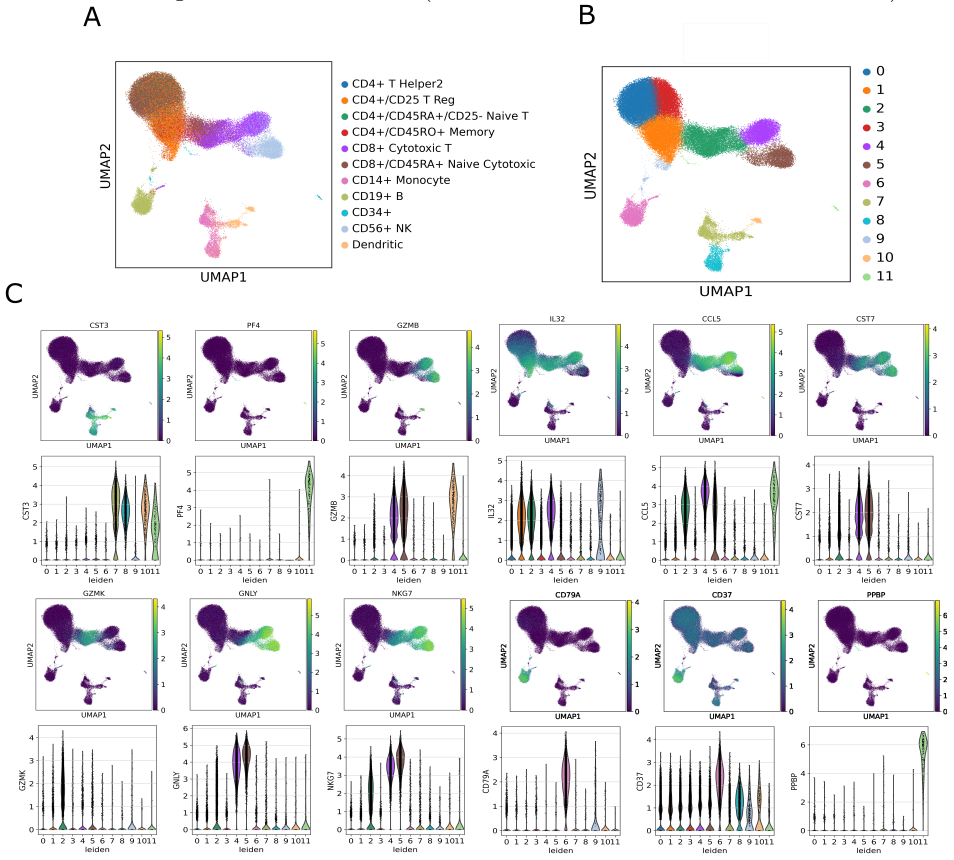

Fig B: Marker analysis using sc-CGconv. After clustering DE genes are identified using clustering of PBMC data with sc-CGconv and results of marker genes on ultra large PBMC datasets. Panel-A. 2D UMAP visualization of PBMC dataset with original cell annotations. Panel-B. 2D UMAP visualization of clustering results with sc-CGconv. Panel-C. visualization of 12 markers which are overlayed based on their expression –low (blue) to high (yellow)- on the reference PBMC UMAP

plot.

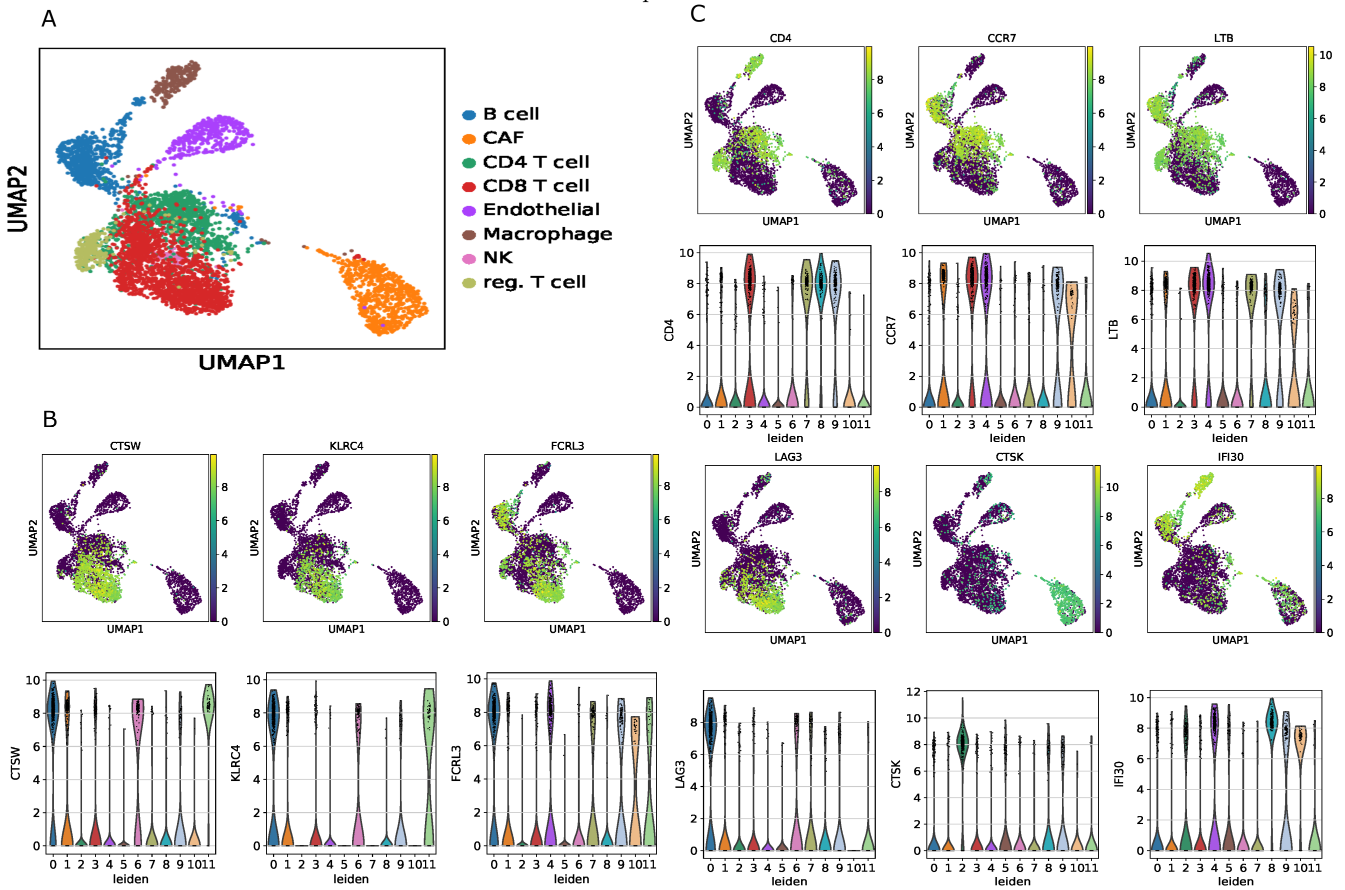

Fig C: The Figure depicts the results of marker gene analysis on melanoma datasets. Panel-A. 2D UMAP visualization of melanoma data with original annotation. Panel B. 2D UMAP visualization of clustering results with sc-CGconv. Panel-C. visualization of 9 markers which are overlayed based on their expression –low (blue) to high (yellow).

References

[1] M. Baron, A. Veres, S. L. Wolock, A. L. Faust, R. Gaujoux, A. Vetere, J. H. Ryu, B. K. Wagner, S. S. Shen-Orr, A. M. Klein *et al.*, "A single-cell transcriptomic map of the human and mouse pancreas reveals inter-and intra-cell population structure," *Cell systems*, vol. 3, no. 4, pp. 346–360, 2016.

[2] A. M. Klein, L. Mazutis, I. Akartuna, N. Tallapragada, A. Veres, V. Li, L. Peshkin, D. A. Weitz, and M. W. Kirschner, "Droplet barcoding for single-cell transcriptomics applied to embryonic stem cells," *Cell*, vol. 161, no. 5, pp. 1187–1201, 2015.

[3] I. Tirosh, B. Izar, S. M. Prakadan, M. H. Wadsworth, D. Treacy, J. J. Trombetta, A. Rotem, C. Rodman, C. Lian, G. Murphy *et al.*, "Dissecting the multicellular ecosystem of metastatic melanoma by single-cell rna-seq," *Science*, vol. 352, no. 6282, pp. 189–196, 2016.

[4] J. Zhang, "Selecting typical instances in instance-based learning," in *Machine Learning Proceedings 1992*. Elsevier, 1992, pp. 470–479.

[5] G. X. Zheng, J. M. Terry, P. Belgrader, P. Ryvkin, Z. W. Bent, R. Wilson, S. B. Ziraldo, T. D. Wheeler, G. P. McDermott, J. Zhu *et al.*, "Massively parallel digital transcriptional profiling of single cells," *Nature communications*, vol. 8, 2017.
