# Supplementary material for: A copula based topology preserving graph convolution network for clustering of single-cell RNA-seq data
Source: PLoS Comput Biol. 2022 Mar 10;18(3):e1009600. doi: 10.1371/journal.pcbi.1009600 (PMC8979455; doi:10.1371/journal.pcbi.1009600)
Supplement: Attachment — Submitted filename: Review of GCN_Plos.pdf [file pcbi.1009600.s002.pdf]

Answer of Reviewer 1

1. Line 35: “The standard approaches for gene selection (or feature extraction) are failed..” Consider rephrasing to: “The standard approaches for gene selection (or feature selection) fail to…”

Answer: Corrected in the Revised Version.

2. Line 37: “Moreover, the exiting approaches…” Typo here. “Moreover, existing approaches...”

Answer: Corrected in the Revised Version.

3. Line 56: “The advantages of sc-CGconv come selecting a new robust equitable …” Consider substituting ‘come’ or use another appropriate phrase.”

Answer: Corrected in Revised Version.

4. Figure 1: Capitalize ‘W’ in figure legend. Also a typo is observed: “adjacencey matrix”.

Answer: Corrected in Revised Version.

5. Line 70: Why is ‘experiments’ italicized?

Answer: Corrected in Revised Version.

6. Line 71: “(ii) the annotation of cells are accurate ...” Replace “are” with “is”

Answer: Corrected in Revised Version.

7. Lines 81 and 96: It is seen that 81 and 96 state “See -A of figure 1” and “See C in Figure 1” whereas Lines 88 and 103 state “See panel-B” and “See panel-D”. Please consider mentioning either panel-A or Figure 1A,1B (whichever is appropriate).

Answer: Corrected as Figure 1A, 1B in Revised Version.

8. Line 84: Consider rephrasing. “… a thousand genes expression...” This sentence could be written as “We choose cells having more than 1000 genes with non-zero expression values…” Also, the authors could provide an explanation of how the structure of the data matrix is preserved. How does LSH achieve that?

Answer: As advised we have now rephrased the sentence in the revised version. We have now provided an explanation of how the structure of the data matrix is preserved using LSH based sampling. Please see the subsection ‘**Structure-aware feature sampling using LSH**’ (page no. 4) of the revised version of the manuscript.

9. Line 96: “A robust equitable correlation measure Ccor”. The manuscript would greatly benefit if the authors provided an overview of the copula correlation concept to a greater extent. How does this method compare to other correlation metrics? A brief literature survey about the usage of this concept is also warranted to drive home the usage of this correlation measure. Which other fields use copula-based correlation and to what extent are they helpful?

Answer: We have now provided a brief explanation and overview about the usage and advantage of the copula correlation measure over the other correlation measure in the revised version of the manuscript. Please see the subsection ‘**Copula based correlation measure (Ccor**)’ (page no. 11) of the revised version of the manuscript.

10. Line 111: “The result of this step is a feature matrices (Fi)..” Grammatical error – “… is a feature matrix (Fi)..”

Answer: Corrected in Revised Version.

11. Line 112: Which network features are inferred here? Unclear.

Answer: The node features are inferred here. The connectivity among the nodes are inferred as a low dimensional embedding using GCN.

12. Line 120: Consider providing an explanation behind the use of Adam optimizer and ReLu as activation functions. A comment on the learning rate for the optimization procedure would be helpful.

Answer: As advised we have now included the rationale for using Adam optimizer and ReLu as activation functions. Here we used ADAM which used adaptive learning procedures in the optimization process. Please see the section “**Training of graph convolution network on cell-cell graph**” (page no -4).

13. Line 122: Manuscript could benefit by the addition of a figure for the ROC curve, in addition to the ROC statistics in table1.

Answer: As advised we have now included the ROC curve (validation ROC) corresponding to table-1 as a supplementary figure-1 of the supplementary text.

14. Table1: Table legend states –“First two columns of the table shows total number of nodes and number of edges..” whereas in the table itself number of edges is written first followed by number of nodes.

Answer: Corrected in Revised Version.

15. Line 125: Wolf et al should be capitalized. Manuscript states "…wolf et al".

Answer: Corrected in Revised Version.

16. Line 126: Consider writing the full name of Kullback-Leibler (KL) divergence since this is the first time

Answer: Corrected in the revised version.`

17. Line 129: "We measured the KL divergenece". Typo here.

Answer: Corrected in Revised Version.

18. Line 132: The correct usage of the phrase is "state of the art" and not "arts". Please rectify. Also in Table 2 (Table caption).

Answer: Corrected in Revised Version.

19. Figure 2: 'KL div' as a short form for KL divergence should be mentioned in the legend.

Answer: Corrected in Revised Version.

20. Line 157: What is the mathematical interpretation of the Clayton Copula? How does the value of theta matter? What is its physical implication towards the performance of scCGconv?

Answer: The clayton copula is a special type of asymmetric archimedean copula which is appropriate for high dimensional datasets to capture the dependence structure. Here \theta defines the generator function of the copula. The degree of dependence is adjusted by varying \theta. For Clayton copula \theta is defined as \theta \in [-1,Inf]/(0). We have now briefly defined the Clayton copula in the subsection **Clayton copula** (page no. 11) of the revised version of the manuscript.

Here we select theta as 0.001 as it gives better performance in our experiments.

21. Line 174: "Once features / (low dimensional embedding) …" Please consider replacing / with or and remove parentheses. It is confusing.

Answer: Corrected in Revised Version.

22. Line 179 and Figure 3 (Legend): What do the authors mean by "..(Kandle tau " in line 179 and "Kandel Tau in Figure 3 legend.."? Do the authors intend to say Kendall tau? Please rectify if this is the case.

Answer: Corrected in Revised Version.

23. Line 180: grammatical error - "…for all competitive method…" should be - "…for all competitive methods…"

Answer: Corrected in Revised Version.

24. Line 182: This section should be considered for rewriting. How exactly are the authors identifying marker genes using their method? A detailed explanation is warranted here.

Answer: We have now rewritten the section to give a detailed explanation about the marker gene selection method. Please see the section "sc-CGconv can identify marker genes" in the revised version .

25. Also line 187 states 19 markers from melanoma and 13 from PBMC dataset are significant; whereas table 3 states 19 and 12. Which numbers are correct?

Answer: It was 12 markers from the PBMC dataset. We have now corrected the typo in the revised manuscript.

26. Line 188: If cell marker is the name of the database, please capitalize the name as in Cell Marker?

Answer: Corrected in Revised Version.

27. Table 3: What do the authors mean by pubmed id of the markers? The numbers in parenthesis do not refer to the NCBI gene id or Ensembl Gene Stable ID. Please provide an explanation of what nomenclature of genes is used here. Also why do all 3 CD8 T cell markers have the same ID i.e. 28622514 although they correspond to 3 different genes? Are these IDs specific to the Cell Marker database used by the authors?

Answer: The pubmed id is an unique identifier specific for every published literature. The pubmed id represents published evidence of the results that supports the existence of the markers for the particular cell type.
The same pubmed id signifies the corresponding published literature has the evidence about the fact that these three genes are the marker of CD8 T cells.
Here we have utilized oficial gene id to represent the gene names.

28. Line 189: What are the markers identified by scCGconv for other 2 datasets (Baron and Klein)? It would be beneficial to have a list of these markers as a Supplementary Table.

Answer: As advised it is now added as Supplementary table-1.

29. Line 209: What is meant by Human Klein and Pollen? Please explain.

Answer: These represent the datasets used here. We have now updated this line to avoid any confusion..

30. In general, how does this method compare to other NN utilizing scRNAseq methods like PHATE and SAUCIE? Please comment on this.

Answer: We have now compared our method with PHATE and SAUCIE. Please see the figure 2 of the revised version of the manuscript.

31. It would be good to see how the list of features selected by this method compares to lists of genes selected by other methods (for example methods that use standardized variance / Fano or Gini index)

Answer: As advised by the reviewer we have now compared the proposed method with Fano and Gini index. Please see table-2 of the revised version of the manuscript.

32. It would also be useful to see an exploration of the properties of genes. For example, Gini picks up genes expressed in few cells, Fano picks up genes with large variance with respect to the mean expression. What are the variational properties of the genes picked up by their method?

Answer: Feature selection within the proposed method is driven by the LSH based sampling method. The selected genes obtained from the bucket of LSH sampling stage are non-redundant and relevant over all the cells. This is ensured by selecting the nearest neighbour within the k-nn graph generated from the samples within one bucket. Moreover, the hash function of the LSH step ensures a structure-aware sampling of the features (genes) over all samples (cells). Thus the genes picked by the proposed method are not only relevant and non-redundant, but preserve the properties of the whole datasets.

33. The authors don't do any intermediate dimensionality reduction (typically one would do feature selection, run PCA, and use the PCs for further computation rather than genes). The construction of the cell kNN graph is done directly in the selected feature space. This could be justified since their gene selection is supposed to remove redundant information, so running PCA might not add much. But this is another place where a formal comparison of the two approaches would be worthwhile to see. PCA and their gene selection method are both essentially trying to remove redundant information; so if one compares their approach with one where there is no feature selection and only PCA (and using PCs for further steps), what does the comparison look like? One example comparison: are the genes that contribute heavily to top PCs in PCA the same (or similar) as the genes retained by their feature selection method?

Answer: We are thankful to the reviewer for raising this point. As advised we have now compared our method by replacing the feature extraction step with PCA. Please see the last paragraph of the subsection 'Comparison with State-Of-The-Art Methods' and table-2 of the revised version of the manuscript.

Answer of Reviewer 2

1. M3Drop is an important and popular feature selection method tailed for scRNA-seq data, the authors also mentioned it for several times in the manuscript. However, there is no comparison with it in Table 2. I would suggest the authors to add it to get a more comprehensive comparison.

Answer: As advised we have now compared our method with M3Drop. Please see Table- 2 of the revised version of the manuscript.

2. As we know, deep learning is a powerful tool, but it often tends to consume a lot of time. To let users fully understand the characteristics of the proposed approach, the authors should provide the running time of all the methods used in this study for all four datasets.

Answer: We have now included a subsection stating the execution time of all the competing methods. Please see the subsection 'Execution time' and Table-3 of the revised version of the manuscript.

3. After I successfully installed the R package "copulaGCN" following the authors' instructions at Github (https://github.com/Snehalikalall/CopulaGCN), running "library(copulagcn)" still got an error. The correct name of the package may be "ucfs"? Please check the package name and modify the correct usage in the README.

Answer: We have now updated the Github page to rectify all the errors. The package is now renamed as sc-GCConv and can be loaded using the library("scGCConv") command in R software.

4. I believe all feature selection methods could easily identify several marker genes, therefore it is not necessary to highlight Table 3. I would urge the authors to put Table 3 in the supplementary files.

Answer: As advised, Table 3 is removed from the main text and given in the supplementary file.

5. In the legend A of Fig1, method "limnorm" does not exist. The authors may refer to "linnorm".

Answer: Corrected in Revised Version.

6. Several important reviews concerning feature selection and clustering of scRNA-seq data are missing, such as:

1) Zhang Z, Cui F, Wang C, Zhao L, Zou Q: Goals and approaches for each processing step for single-cell RNA sequencing data. Briefings in bioinformatics 2021, 22(4).

2) Zhu X, Li H-D, Guo L, Wu F-X, Wang J: Analysis of Single-Cell RNA-seq Data by Clustering Approaches. Current Bioinformatics 2019, 14(4):314-322.

Answer: As advised the references are added in revised manuscript with citation 16 and 9 respectively.
